# Two-photon excitation of FluoVolt allows improved interrogation of transmural electrophysiological function in the intact mouse heart

**DOI:** 10.1016/j.pbiomolbio.2019.08.007

**Published:** 2020-08

**Authors:** Simona Salerno, Karin Garten, Godfrey L. Smith, Tomas Stølen, Allen Kelly

**Affiliations:** a Department of Circulation and Medical Imaging, St. Olav's Hospital, Norwegian University of Science and Technology (NTNU), Trondheim, Norway; b Institute of Cardiovascular & Medical Sciences, University of Glasgow, Glasgow, UK

**Keywords:** Voltage sensitive dyes, Isolated perfused heart, Two photon excitation microscopy, Electrophysiology

## Abstract

**Background & aims:**

Two-photon excitation of voltage sensitive dyes (VSDs) can measure rapidly changing electrophysiological signals deep within intact cardiac tissue with improved three-dimensional resolution along with reduced photobleaching and photo-toxicity compared to conventional confocal microscopy. Recently, a category of VSDs has emerged which records membrane potentials by photo-induced electron transfer. FluoVolt is a novel VSD in this category which promises fast response and a 25% fractional change in fluorescence per 100 mV, making it an attractive optical probe for action potential (AP) recordings within intact cardiac tissue. The purpose of this study was to characterize the fluorescent properties of FluoVolt as well as its utility for deep tissue imaging.

**Methods:**

Discrete tissue layers throughout the left ventricular wall of isolated perfused murine hearts loaded with FluoVolt or di-4-ANEPPS were sequentially excited with two-photon microscopy.

**Results:**

FluoVolt loaded hearts suffered significantly fewer episodes of atrio-ventricular block compared to di-4-ANEPPS loaded hearts, indicating comparatively low toxicity of FluoVolt in the intact heart. APs recorded with FluoVolt were characterized by a lower signal-to-noise ratio and a higher dynamic range compared to APs recorded with di-4-ANEPPS. Although both depolarization and repolarization parameters were similar in APs recorded with either dye, FluoVolt allowed deeper tissue excitation with improved three-dimensional resolution due to reduced out-of-focus fluorescence generation under two-photon excitation.

**Conclusion:**

Our results demonstrate several advantages of two-photon excitation of FluoVolt in functional studies in intact heart preparations, including reduced toxicity and improved fluorescent properties.

## 1 Introduction

The heart is an electrically excitable organ, capable of transducing the rapid and coordinated spread of electrical excitability into cycles of mechanical contraction and relaxation, allowing efficient ejection of blood from the heart and subsequent filling of the chambers in preparation for the next beat. Many cardiac diseases manifest some form of cardiac arrhythmia; an electrical dysfunction often triggered by alterations in membrane ion channel function, structural abnormalities, or impaired calcium handling. Developing better mechanistic insight into the formation and termination of lethal arrhythmias, manifestly a multicellular phenomenon, requires characterization of electrical activity across a large volume of intact cardiac tissue, including the intramural wall.

The gold standard method for membrane voltage measurements is sharp microelectrode recordings, which provide direct and accurate measurements of membrane potentials. However, microelectrode recordings do not allow large-scale tissue mapping. Optical methods are a widely used alternative, offering the advantage of preserving the integrity of the tissue while providing indirect but high-fidelity measurements of membrane potentials from large volumes of intact cardiac tissue. Voltage sensitive dyes (VSDs) have become an indispensable tool to measure rapid electrophysiological signals in excitable biological media. In cardiac preparations, this has allowed real-time mapping of electrical conduction across the heart's surface. Recently, two-photon excitation microscopy using VSDs has improved investigation of the conduction properties of deep regions within intact cardiac tissue. In particular, two-photon microscopy affords the ability to interrogate electrophysiological function with subcellular precision down to an axial depth of 450 and 500 μm in mouse and rabbit myocardium, respectively (Ghouri et al., 2015).

In the context of cardiac electrophysiology, the most important requirement of a VSD is the ability to track a fast event such as the upstroke (phase 0) of an action potential (AP). One category of VSDs that fulfills this requirement is the electrochromic dyes, whose voltage-sensing mechanism is based on a shift in spectral characteristics in response to changes in membrane electric field (Loew, 1996). This property allows recordings of APs with high signal-to-noise ratio (S/N) simply by ratioing the right and left sides of the emission curve, minimizing the distorting effects of motion artifacts, bleaching and uneven distribution of the dye (Loew, 1996). Di-4-ANEPPS is one of the most widely used electrochromic VSDs, capable of a fractional change in fluorescence (ΔF/F) of 10% per 100 mV and a response time in the order of femtoseconds (Loew, 1996). It is often used in the setting of isolated hearts experiments (Ghouri et al., 2015; Dumas and Kinisley, 2005; Kelly et al., 2017). On the other hand, several adverse effects of di-4-ANEPPS have been reported in both isolated cardiomyocytes and intact heart preparations. These include prolongation of the AP duration, occurrence of early afterdepolarizations (Schaffer et al., 1994), slowed atrioventricular (AV) conduction (Nygren et al., 2003), and reduced longitudinal and transverse ventricular conduction velocity in isolated hearts (Larsen et al., 2012).

Recently, another category of VSDs has emerged which records membrane potential by photo-induced electron transfer (PeT), a process whereby a fluorescent reporter is enhanced or quenched by an electron-rich donor through a membrane spanning molecular wire. A change of membrane electric field alters the rate of electron transfer and, in turn, the intensity of the emitted fluorescence (Miller et al., 2012). FluoVolt is a commercially available PeT-based VSD which promises fast response and 25% ΔF/F per 100 mV. The few studies using FluoVolt published to date focus mainly on iPSC-derived cardiomyocyte monolayers (McPheeters et al., 2017; Bedut et al., 2016) and neuronal cell cultures (Pakhomov et al., 2017). These studies have reported a ΔF/F of 12–20% in cardiomyocyte monolayers and 7% in neuronal cell cultures. However, there are no reports of FluoVolt's performance in intact cardiac preparations.

In this study we aimed to characterize the fluorescence properties of FluoVolt following two-photon excitation as well as its utility for deep tissue cardiac imaging. We also demonstrate the ability to simultaneously record calcium transients using a single excitation wavelength in a dual-detector system.

## 2 Methods

### 2.1 Ethics statement

All animal experiments were approved by the Norwegian Council for Animal Research and were conducted in accordance with the Guide for the Care and Use of Laboratory Animals (National Institute of Health, 8th edition, revised 2011).

### 2.2 Excitation/emission characterization of FluoVolt using isolated cardiomyocytes

The emission spectra of FluoVolt and di-4-ANEPPS were obtained with a Leica SP8 STED 3X microscope equipped with a tunable white-light laser, using rat left ventricular cardiomyocytes, isolated as described in detail previously (Kemi et al., 2002). In brief, hearts from female Sprague Dawley rats were removed under 2% isoflurane anesthesia and immediately transferred for cardiomyocyte isolation by retrograde Langendorff perfusion with a modified Krebs solution containing collagenase type II (Worthington, UK). Following isolation, cardiomyocytes were stained with one of the two dyes according to the manufacturer's instructions. The two-photon excitation spectra of the dyes were obtained using a Zeiss LSM 510 microscope (Carl Zeiss, Jena, Germany) coupled to a tunable Ti:Sapphire laser (Mira Model 900–F; Coherent, Inc., Laser Group, Santa Clara, CA, USA). Adherent, fixed HL-1 cells were excited with a range of wavelengths from 700 nm to 1000 nm. For primary cardiomyocyte and isolated perfused heart imaging, a Zeiss Axio Examiner Z1 microscope coupled to a tunable Ti:Sapphire laser (Chameleon Vision-S, Coherent, Santa Clara, CA, USA) mounted on a two-photon microscope system (Intelligent Imaging Innovation, Denver, CO, USA) was used. Emission was collected using a pair of high sensitivity gallium arsenide phosphide (GaAsP) photodetectors at 525–545 nm and 590–650 nm, respectively.

### 2.3 Simultaneous recordings of action potentials and calcium transients in isolated perfused hearts

C57BL/6 male mice (age 10–24 weeks) were anesthetized with a gaseous mixture of 2% isoflurane in 70:30 N_2_O and O_2_ and given an intravenous injection of heparin (1 ml of a 5000-IU solution per kg body weight). The hearts were then excised and retrogradely perfused on a modified Langendorff perfusion system with Tyrode's solution (116 mM NaCl, 18 mM NaHCO_3_, 1 mM MgSO_4_, 5 mM KCl, 1 mM Na_2_HPO_4_, 11 mM Glucose, 1.2 mM Na-pyruvate, 1.4 mM CaCl_2_) at 37 °C. The solution was aspirated with a mixture of 95% O_2_/5% CO_2_ to provide oxygen and maintain pH at 7.4. Flow was adjusted to achieve a coronary perfusion pressure between 60 and 80 mmHg. Following a 10 min equilibration period, perfusion was switched to Tyrode's solution containing blebbistatin (10 μM) and 2,3-butanedione monoxime (10 mM) to inhibit contraction. The hearts were loaded with the voltage-sensitive dyes FluoVolt (15–17  μl in 500 μl of Tyrode's solution containing 150–170 μl pluronic acid, N = 8) or di-4-ANEPPS (25  μL of 2 mmol/l stock solution, N = 9). Concentration of FluoVolt is not specified by the manufacturer. FluoVolt loaded hearts were also loaded with the calcium indicator Rhod 2-AM (50 μl of 1mg/1 mL stock solution). Possible adverse effects due to dye loading were observed in a pseudo-electrocardiogram (ECG) recorded throughout the experiments. ECG traces were analyzed using LabChart analysis modules (ADInstruments, Dunedin, New Zealand). To confirm the accuracy of the measurements, a subset of ECG traces were analyzed manually as previously described (Thireau et al., 2008). QRS duration was measured as shown in Supplementary Fig. 5.

Hearts were electrically paced from the endocardial surface of the left ventricle (LV) at 7 Hz (pacing cycle length of approximately 144 ms) using a Mouse Octapolar Electrophysiological Catheter (Emka Technologies, Paris, France) introduced into the LV via the aortic cannula. The choice of ventricular pacing was justified by the incidence of AV block in di-4-ANEPPS loaded hearts, which made atrial pacing unsuited for obtaining a consistent pacing rate of 7 Hz. The suitability of using endocardial pacing to produce endo-epi patterns of conduction has been verified previously in our lab (Kelly et al., 2017). Discrete tissue layers (spaced 50 μm apart) throughout the LV transmural wall were sequentially line scanned with 840 nm (FluoVolt and Rhod 2-AM loaded hearts) and 950 nm (di-4-ANEPPS loaded hearts) excitation. Electrophysiological signals were acquired with a 20X/1.0 NA water immersion objective. Selected image regions for line scanning were 250–400 μm long, acquired at a rate of 1000–1500 Hz. Line scan images were processed by averaging the pixel values of each line (5000–6000 lines per layer) to produce a 2D data plot of fluorescence intensity with respect to time. From this, 25 individual APs were averaged by pacing cycle length to obtain a single AP from each transmural layer. Exemplary line scan images and corresponding averaged traces are shown in Supplementary Figures 1, 2 and 3 for di-4-ANEPPS, FluoVolt and Rhod 2-AM, respectively.

### 2.4 Two-photon axial beam profile

To estimate out of focus excitation generated by the excitation laser source, the z-axis profile of the two-photon laser was imaged using a fluorescent cuvette method similar to that described previously (Ying et al., 1999). Briefly, a custom-designed, 3D-printed sample cuvette with an objective lens collar was attached to a Zeiss 10X 0.3NA water-immersion objective lens (working distance 3.5 mm). A glass coverslip (thickness 0) was attached to an open side-panel of the cuvette, then filled with a 25  μl sample of either FluoVolt or di-4-ANEPPS diluted in 1.5 ml of deionized H_2_O. The resultant z-axis profile of the two-photon spot generated from this fluorescent sample was then focused onto a CCD camera sensor (Dalsa CA-0256 W, 256^2^ pixels). The collection optics consisted of an Olympus 10X 0.3NA air objective (working distance 10 mm) and a 50 mm tube containing a 650 nm shortpass filter and an f25 planoconvex lens. To estimate the phenomenon of out of focus two photon excitation near the sample surface, the imaging system needed to capture an image plane which encompassed at least the entire working distance of the objective lens. The focusing objective was therefore moved 1.5 mm relative to the imaging objective using the microscope z-drive and two images were captured and stitched together after each experiment. Increasing degrees of out of focus fluorescence were generated by increasing the percentage of scattering beads in the sample cuvette (0.5, 1.0, 1.5 and 2.0%). After images were stitched together, focused and scattered (out of focus) fluorescence was quantified by measuring the fluorescence through a line profile 3 pixels wide (approximate resolution of the 2P beam waist achieved with this imaging set up). Images were stitched together and analyzed using ImageJ and Matlab. A detailed description of the setup, including discussion of imaging limitations can be found in the Supplementary materials and methods.

### 2.5 Chemicals and reagents

Blebbistatin and Rhod2-AM were purchased from Abcam (Cambridge, UK). The FluoVolt membrane potential kit and di-4 ANEPPS were purchased from ThermoFisher (Waltham MA, US). Latex scattering beads were purchased from Sigma (St Louis MO, US).

### 2.6 Data analysis and statistics

Excitation and emission spectra were reconstructed from mean fluorescence intensity values with increasing excitation/emission wavelengths using ImageJ. Two-photon data were analyzed using custom written software in Matlab (Mathworks, Natick, MA, USA). The time from the start of the stimulus to the 10% point of the AP rising phase was used as an index of activation time at each transmural layer. AP duration (APD) was calculated at 50, 75 and 90% repolarization level. ECG traces were acquired and analyzed using LabChart6 (ADInstrument, Oxford, UK) and Matlab. Data are displayed as mean ± standard error. Statistical analysis was performed in Graphpad7 (La Jolla, CA, USA) using Student's *t*-test (paired when appropriate). P-values < 0.05 were considered significant.

## 3 Results

### 3.1 Spectral characterization of FluoVolt

**Fig. 1:**
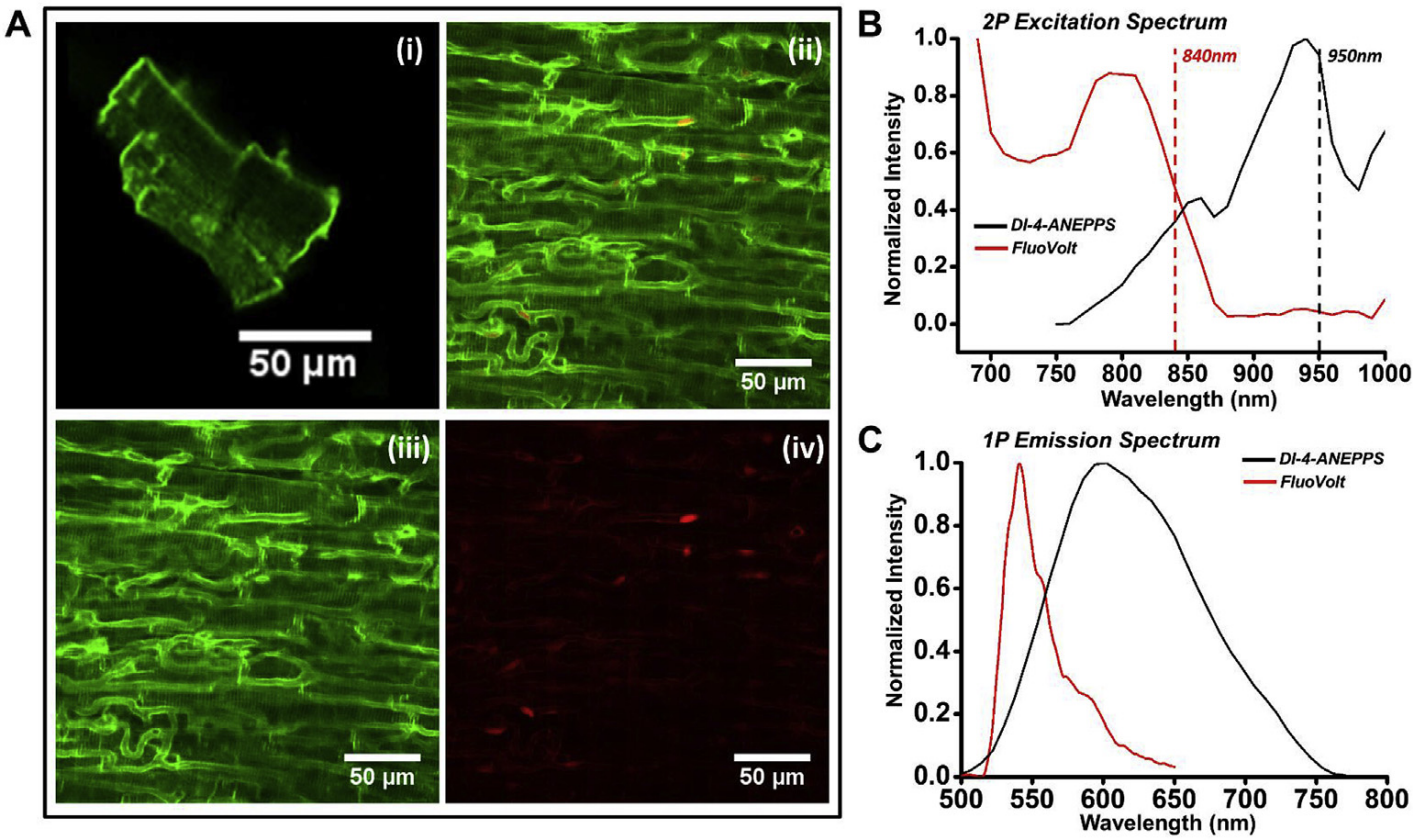
**Spectral characterization of FluoVolt. (A)** Two-photon excitation images of **(i)** an isolated rat ventricular cardiomyocyte stained with FluoVolt and **(ii)** LV murine subepicardial tissue stained with **(iii)** FluoVolt and **(iv)** Rhod-2AM. **(B)** Two photon (2P) excitation spectra of FluoVolt (RED) and di-4-ANEPPS (BLACK). Dashed lines indicate excitation wavelength used for in isolated hearts experiments. **(C)** One photon (1P) emission spectrum of FluoVolt and di-4-ANEPPS.

We began by characterizing the two-photon excitation and emission spectra of FluoVolt. Two-photon excitation images of a FluoVolt stained isolated cardiomyocyte and left ventricular epicardium stained with FluoVolt and Rhod-2AM are shown in Fig. 1A (i) and (ii), respectively. FluoVolt and Rhod-2AM were excited simultaneously at 840 nm and the respective images are given in Fig. 1A (iii) and (iv). 2D images at progressively increasing depths in di-4-ANEPPS- and FluoVolt-loaded hearts are given in Supplementary Fig. 4.

Fig. 1B shows the two-photon excitation spectra of FluoVolt and di-4-ANEPPS. The excitation peak was found at 790–800 nm and 940 nm for FluoVolt and di-4-ANEPPS, respectively. The dashed lines indicate the wavelengths that were used to excite the dyes in the experiments reported in this study. The two-photon excitation spectrum for FluoVolt did not resemble the dual-peak excitation shown for one-photon excitation, published previously (Woodford et al., 2015), which may have predicted a much larger peak around 1000 nm. Fig. 1C shows the emission spectrum of FluoVolt and di-4-ANEPPS, measured with single photon confocal microscopy.

### 3.2 FluoVolt loading is well tolerated by the murine heart

**Fig. 2:**
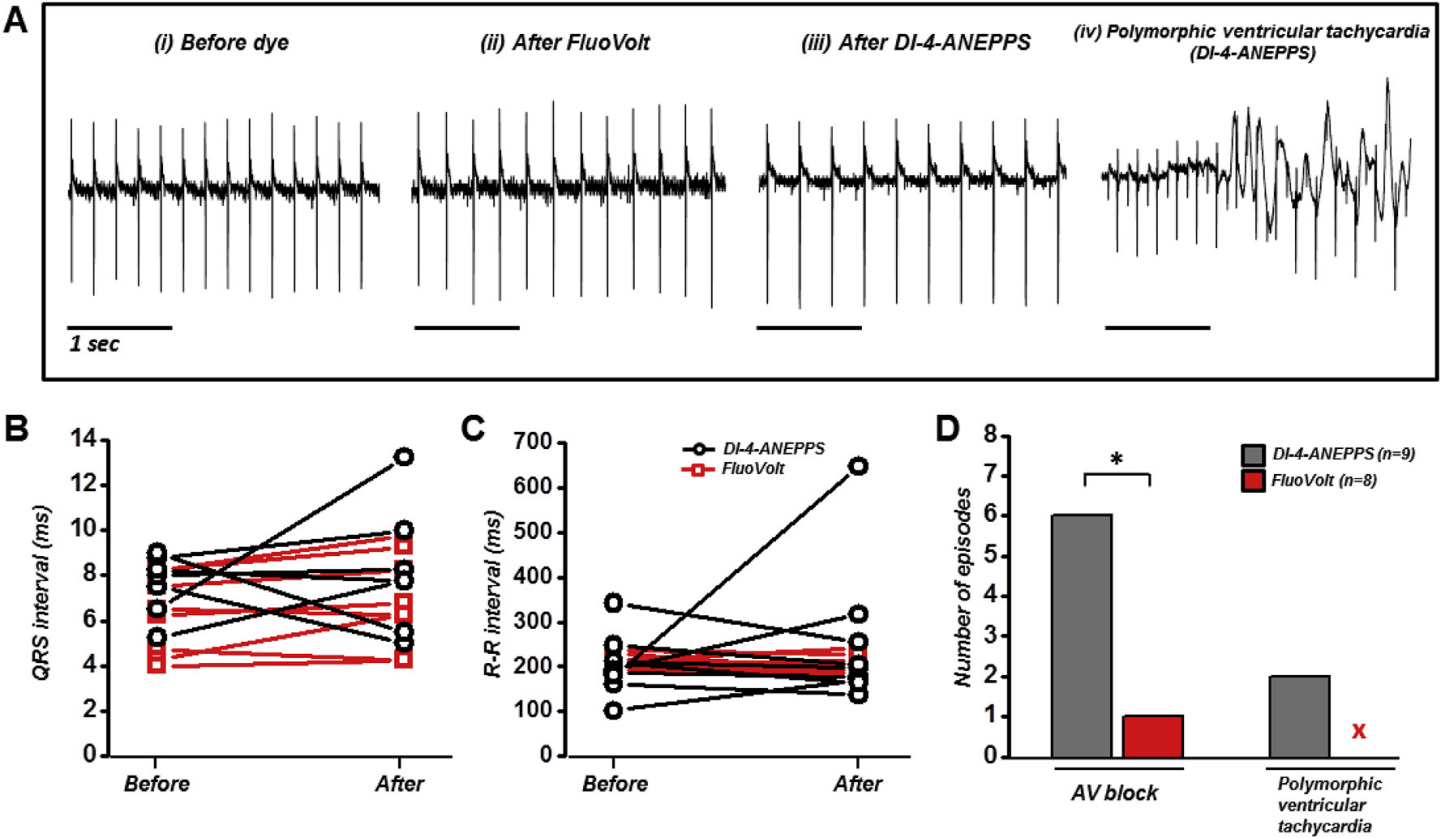
**Whole heart electrophysiology during dye loading. (A)** Representative pseudo ECG traces of isolated hearts **(i)** before dye loading, **(ii)** after FluoVolt loading, **(iii)** after di-4-ANEPPS loading, and **(iv)** example trace of polymorphic ventricular tachycardia caused by di-4-ANEPPS. **(B)** QRS complex and **(C)** R-R interval in individual hearts loaded with FluoVolt or di-4-ANEPPS. **(D)** Binary incidence of AV block and polymorphic ventricular tachycardia (VT) > 10s induced by FluoVolt or di-4-ANEPPS loading. *P < 0.05, Fisher's exact test.

The effect of FluoVolt on normal electrical conduction was observed using pseudo-ECGs recorded during isolated heart perfusion and compared to di-4-ANEPPS. Fig. 2A(i-iii) shows representative ECG traces recorded before and immediately following the cessation of dye loading. Corresponding single cycles of ECGs at expanded time scale are given in Supplementary Fig. 5. Dye loading was approximately 25 min long for di-4-ANEPPS and 12 min long for FluoVolt. No significant difference was found in the QRS duration (Fig. 2B) or R-R interval (Fig. 2C) in either group. QT intervals could not be measured consistently because the pseudo-ECG electrode configuration did not consistently detect an obvious T wave (Supplementary Fig. 5), partly due to the volume of conducting solution in the tissue bath. During dye loading, transient or permanent episodes of atrioventricular (AV) block were detected in 6 of 9 di-4-ANEPPS loaded hearts, while only 1 of 8 FluoVolt loaded hearts suffered from a transient episode of AV block, and no permanent AV block. The observed AV block varied between hearts, with the most common type being second degree AV block (2:1 or 3:1). Transient episodes (>10s) of polymorphic ventricular tachycardia (VT) were also detected in 2 of 9 di-4-ANEPPS loaded hearts (Fig. 2Aiv) and in none of the FluoVolt loaded hearts. The incidence of AV block and polymorphic (VT) events was assessed by visual inspection, and is summarized in Fig. 2D.

### 3.3 FluoVolt-recorded AP signals displayed high dynamic range

**Fig. 3:**
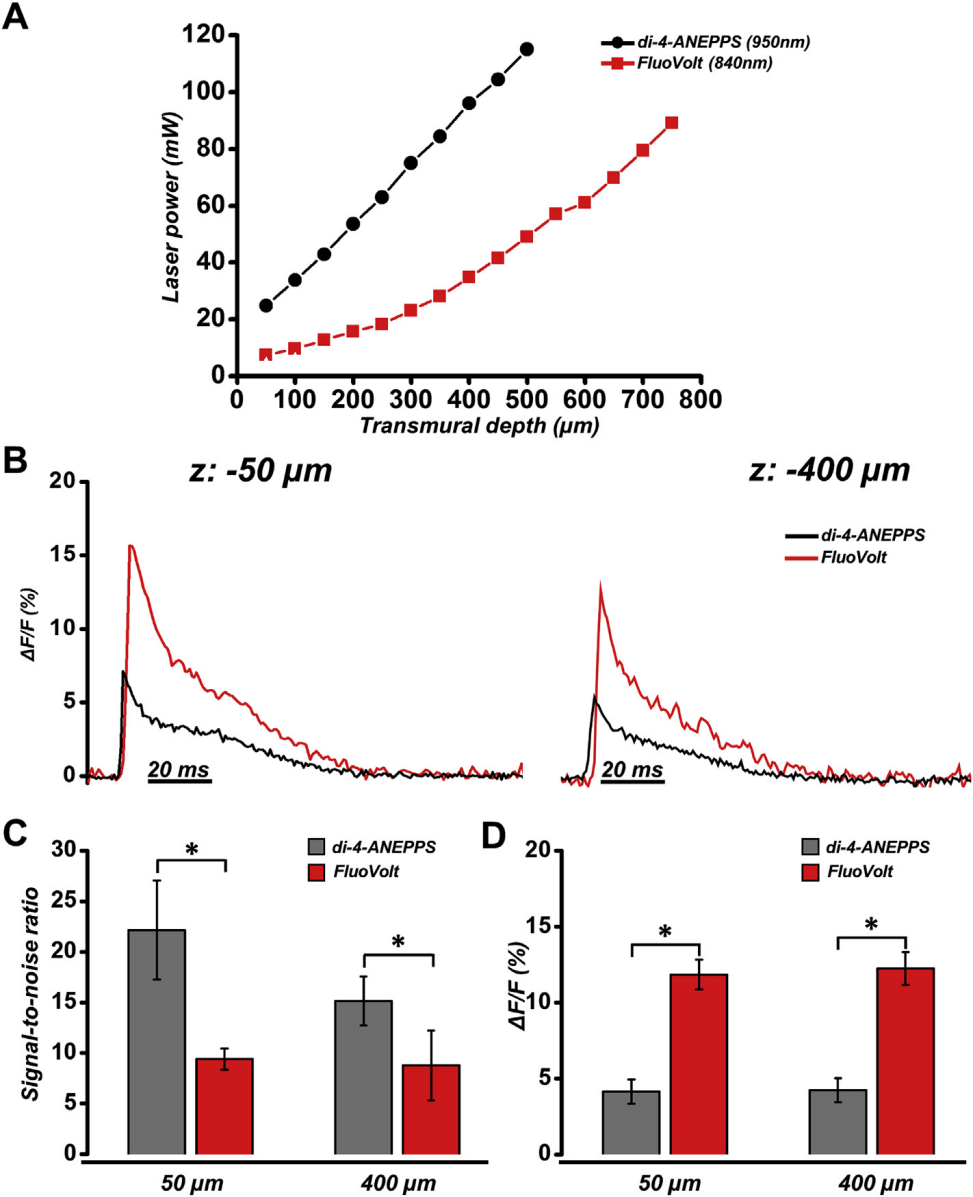
**Fluorescence properties of FluoVolt and di-4-ANEPPS. (A)** Transmural laser power curves for FluoVolt and di-4-ANEPPS. **(B)** Representative superimposed averaged AP traces recorded with FluoVolt and di-4-ANEPPS at 50 μm **(*left*)** and 400 μm **(*right*)** below the epicardial surface. **(C)** S/N of FluoVolt and di-4-ANEPPS signals at 50 μm and 400 μm deep. **(D)** Percentage change of fluorescence in APs recorded with FluoVolt and di-4-ANEPPS at 50 μm and 400 μm deep. *P < 0.05, Student's t-test (unpaired).

After fluorophore loading, we used two-photon microscopy to excite discrete LV transmural layers. Due to increasing scattering and absorption of excitation light at larger transmural depths, excitation power must be increased at each transmural layer to compensate for loss of excitation power. The laser power was therefore adjusted with increasing depth as shown in Fig. 3A, while PMT gain was fixed throughout. To ensure adequate signal-to-noise to measure an AP with each fluorophore at each transmural layer studied, separate transmural power curves were created for both di-4-ANEPPS and FluoVolt. At each layer, less power was used to excite FluoVolt compared to di-4-ANEPPS, despite FluoVolt excitation at shorter wavelengths. Representative superimposed FluoVolt and di-4-ANEPPS AP profiles at 50 μm and 400 μm are given in Fig. 3 left and right panels, respectively. Fig. 3C shows that the signal-to-noise (S/N) ratio of FluoVolt was significantly lower than di-4-ANEPPS both at 50 μm and 400 μm deep (50 μm: 22.14 ± 4.89 *vs* 9.40 ± 1.06, di-4-ANEPPS *vs* FluoVolt; 400 μm: 15.16 ± 2.42 *vs* 8.78 ± 3.46, di-4-ANEPPS *vs* FluoVolt). The dynamic range of the two dyes was also compared at 50 μm and at 400 μm deep. ΔF/F of FluoVolt was significantly larger than di-4-ANEPPS both at 50 μm and at 400 μm of depth (50 μm: 4.15 ± 0.79% vs 11.86 ± 0.99%; di-4-ANEPPS *vs* FluoVolt; 400 μm: 4.25 ± 0.79% vs 12.26 ± 1.09%, di-4-ANEPPS vs FluoVolt, Fig. 3D).

### 3.4 Transmural activation characteristics

**Fig. 4:**
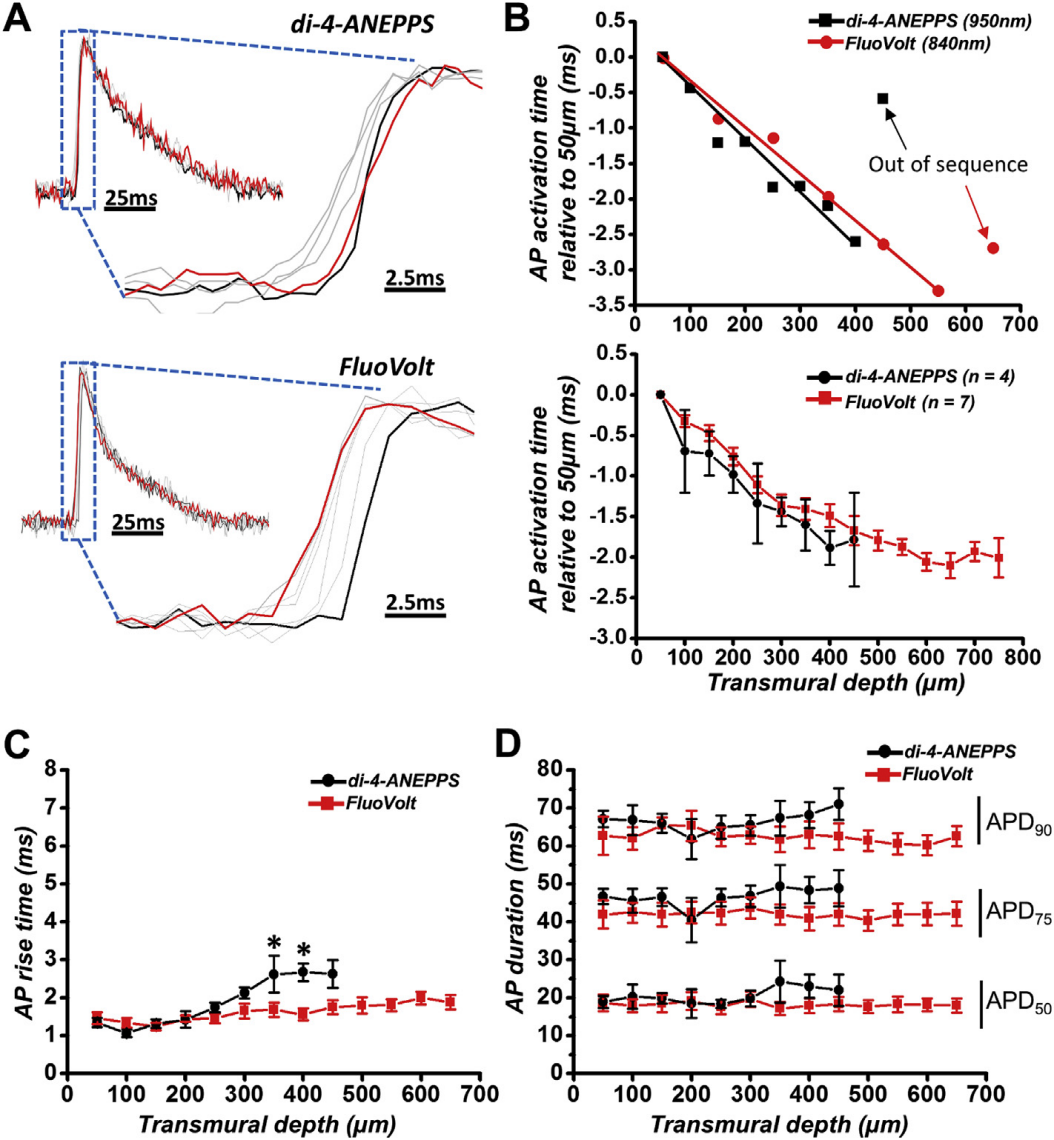
**Transmural cardiac electrophysiology using FluoVolt. (A)** Representative AP traces from sequential transmural layers in isolated hearts loaded with di-4-ANEPPS (upper panel) or FluoVolt (lower panel). Hearts are paced from the endocardial surface to generate endo-epi conduction patterns. Red AP profiles indicate the deepest transmural layer, black AP profiles indicate surface layers, and grey AP profiles indicate intermediate layers. **(B) *Top panel* -** Activation timing of each layer shown in (A) relative to the surface layer. Deepest layers for both dyes are eventually out of sync with the endo-epi activation pattern. ***Bottom panel -*** Average activation timing relative to surface layer of FluoVolt (n = 7) and di-4-ANEPPS (n = 4) loaded hearts. **(C)** Average 10–90% upstroke rise time and **(D)** AP duration (APD) at 50, 75 and 90% repolarization obtained from each transmural layer investigated. *P < 0.05, Student's unpaired *t*-test.

**Fig. 5:**
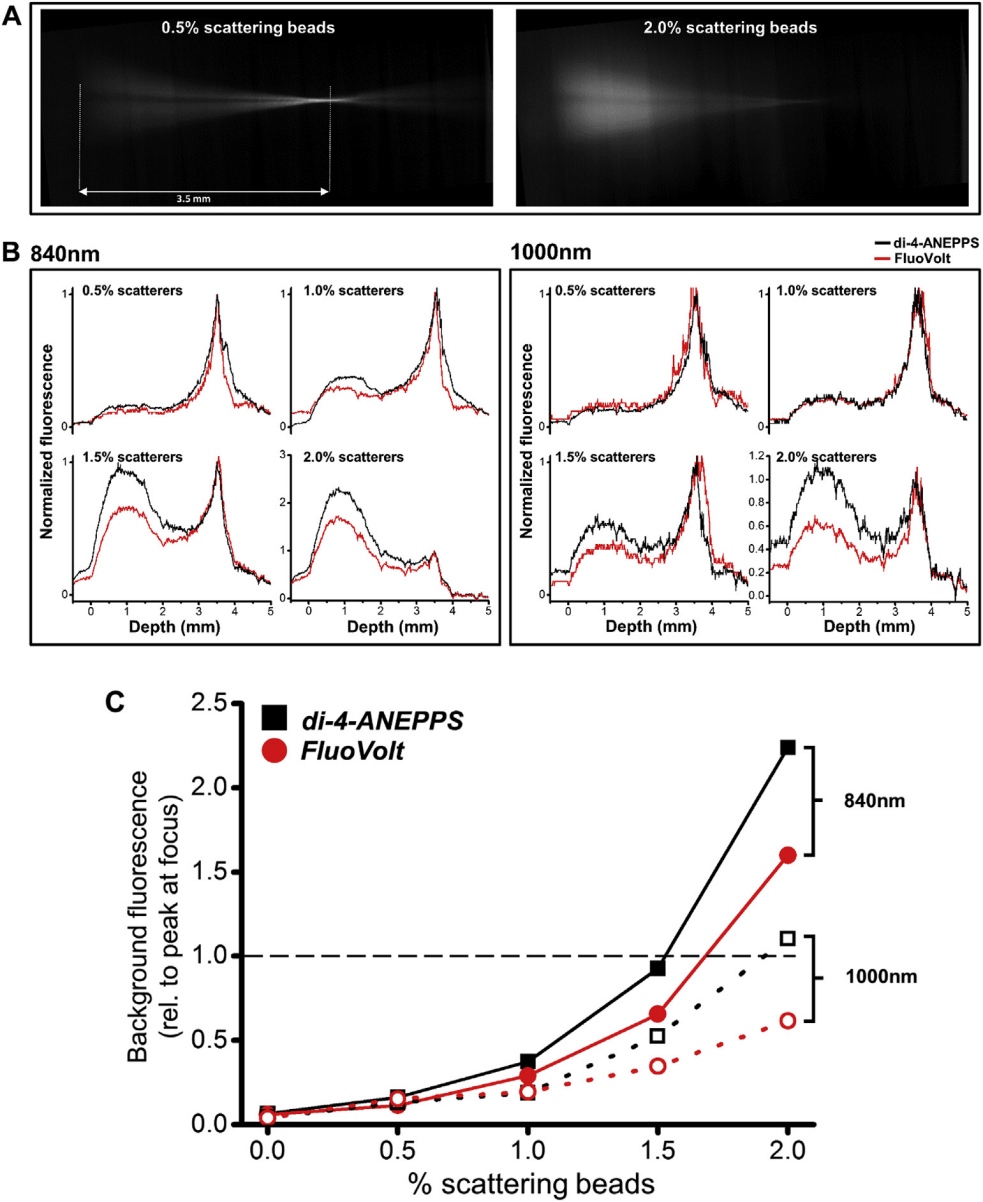
**Z-axis two photon axial beam profile. (A)** Example images of two photon axial beam profile in fluorescent solution containing 0.5% (left) and 2.0% (right) scattering beads. **(B)** FluoVolt and di-4-ANEPPS axial beam profiles at 840 nm (left box) and 1000 nm (right box) excitation wavelength, using increasing concentrations of scattering beads. **(C)** Out-of-focus (background) fluorescence relative to perifocal fluorescence for di-4-ANEPPS and FluoVolt using 840 nm (solid lines) and 1000 nm (dotted lines) excitation. Dashed black line indicates threshold where background dominates and two photon image contrast is lost.

Transmural conduction is a key aspect of cardiac electrophysiology. Despite the sequential nature of the transmural layer interrogation using two-photon microscopy, when the heart is paced an average activation delay profile can be constructed, allowing average conduction velocity to be estimated over a limited range of depths. The AP activation time was measured for each transmural layer as the time delay between the stimulus pulse and the AP upstroke. Fig. 4A illustrates the activation time of subsequent transmural layers in representative APs for an isolated heart loaded with di-4-ANEPPS (upper panels) or FluoVolt (lower panels). APs in red indicate the deepest transmural layer, black indicates the surface layers, and the grey indicates intervening layers. Fig. 4B illustrates the activation timing (relative to the uppermost tissue layer) of each layer from the APs in Fig. 4A, revealing an activation time plot that can be fit with a linear relationship down to 400 μm for di-4-ANEPPS. The next AP layer then appears to activate later (labelled with black arrow). For FluoVolt however, a linear relationship was seen down to 550 μm before the next layer was out of sequence (red arrow). Fig. 5C shows the average behavior of hearts loaded with di-4-ANEPPS (N = 4) and FluoVolt (N = 7). The transmural activation appeared to be linear above 450 μm of depth in di-4-ANEPPS loaded hearts and above 650 μm of depth in FluoVolt loaded hearts (Fig. 4B – lower panel). At 450 μm, the timing of activation was unpredictable for individual di-4-ANEPPS loaded hearts, occurring either earlier or later than at 400 μm (as indicated by the large increase in standard error of the measurements). In FluoVolt loaded hearts, the standard error of the measurements at the deepest layers was smaller compared to di-4-ANEPPS loaded hearts. However, the activation time at 700 μm occurred later than at 650 μm and 600 μm in all hearts.

### 3.5 AP characteristics

AP traces recorded from each transmural layer were averaged in order to remove noise and analyze AP characteristics from a single trace. AP rise time was not different between di-4-ANEPPS and FluoVolt until the deeper transmural layers (350–400 μm, Fig. 4C), however AP duration (Fig. 4D) was not different throughout the myocardial wall at any transmural layer. FluoVolt loaded hearts were also loaded with calcium indicator Rhod 2-AM to demonstrate the possibility of simultaneous recordings of APs and calcium transients. Rhod 2 was excited with the same wavelengths as FluoVolt (840 nm). FluoVolt and Rhod 2 emissions were collected by two separate PMTs at 525–545 nm and 590–650 nm, respectively. A representative AP and calcium transient are superimposed in Supplementary Fig. 6.

### 3.6 Two-photon axial beam profile: di-4-ANEPPS vs FluoVolt

To understand why we could measure average transmural activation deeper using FluoVolt compared to di-4-ANEPPS, we investigated the axial two-photon laser beam profile using controlled scattering samples. In order to visualize the two-photon beam profile, the Ti:Sapphire beam was focused through a fluorescent solution containing either FluoVolt or di-4-ANEPPS, and increasing concentrations of scattering beads. This was imaged onto a CCD camera sensor by an objective and plano-convex lens orientated at right angles to the container with the fluorescent sample. A schematic of the imaging setup is shown in Supplementary Fig. 7A. The beam was focused at 3.5 mm from the interface between the objective lens front aperture and the solution (the working distance of the beam focusing objective lens). This is illustrated in Supplementary Fig. 7B and Fig. 5A.

With 840 nm excitation wavelength (Fig. 5B left) and 0.5% scattering beads, the two-photon axial beam profiles of FluoVolt and di-4-ANEPPS overlapped and a fluorescence peak was obtained at the focus. When the concentration of scattering beads was increased to 1.0%, out of focus fluorescence was detected with both VSDs, as indicated by the appearance of a dome in the beam profile of both VSDs closer to the surface of the solution. When the scattering beads are increased to 1.5%, the amplitude of the dome in the di-4-ANEPPS profile dominates the amplitude of the focus peak. In the FluoVolt beam profile, although the amplitude of the dome increased, the focus fluorescence was still dominant. Finally, when the scattering beads were increased to 2.0%, the amplitude of the dome exceeded that of the focus in both beam profiles.

When the measurements were repeated using 1000 nm excitation wavelength, a similar tendency was observed (Fig. 5B right). The beam profiles of di-4-ANEPPS and FluoVolt overlapped with 0.5% and 1.0% scattering beads. With 1.5% scattering beads, the out of focus fluorescence in the di-4-ANEPPS profile was more pronounced than in the FluoVolt profile, although the focus fluorescence dominated in both profiles. With 2.0% scattering beads, the out of focus fluorescence equalled the focus fluorescence only in the di-4-ANEPPS profile, while in the FluoVolt profile focus fluorescence was still dominant. This data is summarized in Fig. 5C, demonstrating that out-of-focus fluorescence for a given concentration of scattering beads is consistently lower for FluoVolt, independent of wavelength.

## 4 Discussion

FluoVolt is a novel VSD commercialized as a kit optimized for single cell experiments. In this study we attempted to characterize FluoVolt in the intact murine heart using two-photon microscopy. FluoVolt exhibited lower toxicity, higher dynamic range, and lower two-photon excitation power requirements than a frequently-used alternative, di-4-ANEPPS. In addition, FluoVolt generated less out of focus fluorescence than di-4-ANEPPS in highly scattering media. This has the fortuitous effect of allowing electrophysiological signals to be recorded from deeper ventricular transmural tissue layers than has been possible before.

Two-photon excitation spectra do not always coincide with exact two-fold single-photon excitation spectra, but they are often blue-shifted by ∼10–20 nm (Stobrawa et al., 2002). Fluorescence emission on the other hand is independent of the type of excitation (Stobrawa et al., 2002). Therefore, the emission spectrum of FluoVolt was characterized with single-photon confocal microscopy. The measured emission peak was 540 nm, slightly red-shifted relative to the manufacturer's specifications. The two-photon absorption (TPA) maxima between 700 and 900 nm represents an apparent peak well below the predicted TPA maxima for FluoVolt (∼1080 nm) based on the single-photon spectrum (Miller et al., 2012). Indeed, rather than increasing fluorescence as excitation wavelength approached 1000 nm, the fluorescence intensity reached a minimum around 860 nm with FluoVolt and did not appear to increase again (Fig. 1C). This behavior has been observed previously with both Fluo-3 and Rhod-2, although the mechanism remains unclear (Smith et al., 2010).

### 4.1 FluoVolt exhibits multiple favourable characteristics for intact tissue imaging

In the setting of an isolated perfused heart, time is a crucial factor for the viability of the preparation and consequently reproducibility of the data. The time needed for sufficient loading of FluoVolt was shorter compared to di-4-ANEPPS when it was found that toxicity was less of an issue. This allowed us to slightly increase the time window for actual data recording. Despite FluoVolt being injected in a bolus of larger volume and in a shorter time, FluoVolt-loaded hearts suffered significantly fewer episodes of AV block and no episodes of polymorphic ventricular tachycardia compared to di-4-ANEPPS-loaded hearts. These observations suggest that FluoVolt is well tolerated by the murine heart. On the other hand caution may still be warranted, as evidence of phototoxicity of FluoVolt following high excitation illumination has been observed in single cardiomyocytes when using the dosage indicated by the manufacturer (Bedut et al., 2016). The confined two-photon excitation in larger, well-coupled tissue may potentially mask this effect in the intact heart. Restricting excitation time to 10s may also limit any phototoxic effects.

Optimal VSDs must meet several key criteria to be effective for biological applications: as well as low cellular and phototoxicity, VSDs must be bright, resistant to bleaching, respond rapidly and exhibit near linear sensitivity across a broad range of physiological membrane potentials (Miller et al., 2012). While no one class of dye fits perfectly into each category, FluoVolt and other PeT derivatives exhibit many favourable characteristics, including large ΔF/F (between 16 and 30%) (Woodford et al., 2015), linearity over a wide range of physiological membrane potentials (−100 to +100 mV) and response rates in the μs time range (Miller et al., 2012; Woodford et al., 2015), which makes them a suitable option for measuring cardiac APs. The functional mechanism of PeT dyes does not allow the exploitation of ratiometry to increase the S/N ratio, and our results evidence that FluoVolt emission has a significantly lower S/N ratio compared to the ratiometric dye di-4-ANEPPS. However, the lower S/N ratio is compensated somewhat by a larger ΔF/F. In addition, much less excitation power was required for FluoVolt to achieve approximately uniform S/N throughout the transmural layers interrogated.

Optically measured APs are generally characterized by a lower S/N compared to electrically measured APs. Nevertheless, in this study AP characteristics were similar when measured with FluoVolt and di-4-ANEPPS and consistent with microelectrode recordings reported in a similar setting (Knollmann et al., 2001). Sharp microelectrode techniques are advantageous in that they provide a direct measure of cell membrane potential, with high signal bandwidth (20 kHz+) and signal to noise ratios, allowing fine details of individual APs to be resolved. Optical methods by comparison are limited by a combination of the light-collection capability of the optics and the speed of the scanning system (∼2 kHz for raster scanning, 8–10 kHz for resonant scanning). Optical methods excel vs microelectrode techniques in their ability to deliver higher throughput data and, as in this study, technically easier transmural measurements which avoid physical disruption of intact tissue. The AP measurements in this study did not reveal transmural gradients of repolarization, which are often reported in the literature (Wen et al., 2018; Nabauer et al., 1996; Litovsky and Antzelevitch, 1988). This can be explained by the type of preparation, since electrotonic interactions in a well-coupled myocardium are thought to mask repolarization gradients, especially in small hearts (Sampson and Henriquez, 2005). In addition, the deepest recordings made with the current two-photon microscope setup (600–650  μm) represent only a fraction of the entire thickness of the mouse LV (1.5–2 mm thick). Previous reports from our lab using the rabbit left ventricular wedge preparation have shown transmural APD gradients of only 2–4 ms over a similar range of depths (Myles et al., 2010), well within the resolution of the current two-photon measurements. Taken together, it is unlikely repolarization gradients would be present over such a small region and may be less pronounced in well-coupled intact tissue.

### 4.2 Optimising two photon microscopy for cardiac deep tissue imaging

In this study we optically tracked the transmural activation of the LV myocardium using FluoVolt or di-4-ANEPPS using sequential intramural line scan recordings, similarly to previous studies (Kelly et al., 2017). The ability of two-photon excitation to limit excitation to a defined volume within the plane of focus of the imaging system allows close to diffraction-limited imaging with inherent optical sectioning. Techniques to enhance light collection such as lower numerical aperture (NA) objective lenses to increase the solid angle of collection (Tung et al., 2004; McMullen and Zipfel, 2010; Singh et al., 2015; Combs et al., 2011) and light collection in the transmission plane (Dvornikov and Gratton, 2016) can be employed to take advantage of the fact that the scattered emission arises mainly from the focal plane. Enhancements to the excitation characteristics include imaging within the second infra-red imaging window (>1000–1300 nm) and using regenerative amplifiers to increase laser pulse energy at lower repetition rates. Individually, such tweaks have made two-photon imaging down to 1 mm deep in highly scattering brain tissue routine for current commercially available microscope systems. Indeed, in vivo two-photon deep tissue imaging in mouse cortex has been demonstrated down to a depth of 1.6 mm using a highly optimized microscope with 1280 nm excitation (Kobat et al., 2011).

It has been recognized for some time however that a fundamental depth limit exists with two-photon imaging, which has been described numerically (Theer and Denk, 2006; Young et al., 2011) and experimentally (Ying et al., 1999) previously. This can be quantified for a given scattering sample by the ratio of perifocal to out-of-focus fluorescence, with the limit being reached at a depth where this ratio is equal to 1. Factors affecting the generation of out-of-focus fluorescence include the temporal distribution and effective NA of the excitation light, the scattering properties and staining of the sample (Theer and Denk, 2006), and spherical aberration (Young et al., 2011). Approaches to reduce out-of-focus fluorescence and image beyond the fundamental depth limit include use of deformable mirrors to defocus the excitation beam, quenching focal fluorescence and leaving only the out-of-focus fluorescence, which is subtracted from the original image (Leray et al., 2008), and line scanning temporal focusing microscopy (Zhang et al., 2018). Substantial gains could in theory also be possible by using even shorter laser pulses (<50fs) (Theer and Denk, 2006). These approaches are associated with significant cost and complexity of implementation, potentially leaving them out of reach for non-specialist users. In the current study, using a previously published experimental approach combined with our whole heart experimental results, we have shown that FluoVolt allows electrophysiological recordings from deeper tissue layers than di-4-ANEPPS, most likely because less out-of-focus fluorescence is generated for a given degree of sample scattering with FluoVolt. This implies that with increasing depth, as the point spread function blooms, the three dimensional resolution is gradually lost, and more than a single myocardial layer is expected to be excited. Interestingly, this effect appears to be dependent on the dye used to stain the preparation. This finding raises several important questions. Firstly, it may be possible to design the chemical structure of a fluorophore specifically for deep tissue imaging. Chemical engineering strategies for optimising the non-linear absorption characteristics of fluorophores concentrate on enhancing two photon action (TPA) cross section (Pawlicki et al., 2009). The reduced out-of-focus fluorescence generated by FluoVolt is unlikely to represent an enhanced TPA cross section relative to di-4-ANEPPS however. A recent publication by the Miller group demonstrated that FluoVolt in fact has a relatively average two-photon excitation cross section (Kulkarni et al., 2017). The observation that fluorophores can potentially generate different levels of out-of-focus fluorescence in highly scattering media is an important concept when considering fluorophore choice for two-photon deep tissue imaging and warrants further investigation.

A second important aspect relates to the interpretation of electrophysiological signals from cardiac muscle using deep tissue imaging approaches such as two-photon microscopy. Resolving deep structures in live, intact cardiac muscle is restricted by the highly light scattering nature of the tissue. This limits structural information to the first ∼300  μm below the epicardium. Electrophysiological signals, however, require less signal to resolve, have a lower axial constraint than structural features (one cardiac myocyte width (∼25–30  μm) vs 1–2 μm for structural features) and are less distorted by residual motion artifact. As such, we have demonstrated previously using di-4-ANEPPS that fluorescent membrane potentials can be recorded down to 400–450  μm in mouse and rat (Ghouri et al., 2015; Kelly et al., 2017), and 600  μm in rabbit (Kelly et al., 2013). The reason for species-specific variation is not known but is likely related to the cellular content difference between species; while cardiac myocyte size is relatively conserved between mammalian species of different size, the relative volume of mitochondria - an optically dense organelle – is higher in rat and mouse vs rabbit. This fundamental depth limit only becomes apparent when considering the direction and timing of a signal with a rapid component, such as an action potential. Performing a line scan with the microscope objective focus at >450  μm deep in a well-loaded sample results in the return of a measurable signal. Indeed, in the current study we were able to record APs beyond the identified depth limit. However, the timing of the signal relative to the direction of wavefront travel (in this case endocardium to epicardium) allowed us to identify those signals as arising from layers above the objective plane of focus (closer to the epicardium). This would not have been possible using calcium transients, which have a much slower time course and do not reflect direct electrical activation of the myocyte. Caution is therefore warranted when recording deep tissue electrophysiological signals from intact cardiac muscle without some means of identifying the depth limit.

### 4.3 Combined voltage and calcium imaging

Here we demonstrated that FluoVolt could be combined with the calcium indicator Rhod-2AM to achieve simultaneous optical recording of voltage and calcium in a 2-detector setup using a single excitation wavelength. Rhod-2 was bolus-loaded in the current study, rather than being introduced as a dilution in Tyrode's solution over a longer (15 min) period, as has been described previously (Rubart et al., 2003). In addition, laser power was adjusted relative to the FluoVolt signal, rather than to Rhod-2. These factors may have contributed to the relatively low signal-to-noise ratios of the recorded calcium transients. Rhod-2 has some caveats associated with it's use. In particular, a relatively low Ca^2+^ affinity (∼570 nM) and a tendency to compartmentalize within mitochondria due to its net positive charge may limit its applicability to some studies (Paredes et al., 2008). Other more suitable calcium indicators may potentially be paired with FluoVolt for two-photon imaging. The red-shifted Calcium Ruby family of indicators show relatively broad two-photon excitation profiles, with emission peaks >600 nm, and do not exhibit the same compartmentalization issues as Rhod-2 (Collot et al., 2012). It should be noted that while the peak emission of the Ca-Rubies is around 900 nm a significant two-photon absorption (30–40% of peak) is still evident at 840 nm, which may be sufficient to allow effective pairing with FluoVolt and warrants further investigation.

## 5 Conclusions

Here we describe the use of FluoVolt in the intact murine heart using two-photon microscopy. FluoVolt displayed several beneficial characteristics for whole heart deep tissue electrophysiological measurements when compared to the ratiometric voltage sensitive fluorophore di-4-ANEPPS, including low cytotoxicity, fast response times indistinguishable from di-4-ANEPPS and high dynamic range. Simultaneous voltage and calcium imaging was also possible using single wavelength two-photon excitation on a two-detector system in combination with Rhod2-AM. Critically, when imaging throughout the myocardial wall, FluoVolt allowed the excitation of deeper regions in comparison to di-4-ANEPPS, resulting from less out-of-focus fluorescence being produced by FluoVolt for a given imaging depth. This raises the prospect of a voltage sensitive probe design that is optimized to produce less out-of-focus fluorescence in two-photon imaging applications and warrants further study. Overall, our results show that FluoVolt is an attractive probe for electrophysiological studies in isolated perfused hearts.

## References

[bib1] Bedut S., Seminatore-Nole C., Lamamy V., Caignard S., Boutin J.A., Nosjean O., Stephan J., Coge F. (2016). High-throughput drug profiling with voltage- and calcium-sensitive fluorescent probes in human iPSC-derived cardiomyocytes. Am. J. Physiol. Heart Circ. Physiol..

[bib2] Collot M., Loukou C., Yakovlev A.V., Wilms C.D., Li D., Evrard A., Zamaleeva A., Bourdieu L., Mallet J. (2012). Calcium Rubies : a family of red-emitting functionalizable indicators suitable for two-photon Ca^2+^ imaging. J. Am. Chem. Soc..

[bib3] Combs C.A., Smirnov A., Chess D., McGavern D.B., Schroeder J.L., Riley J., Kang S.S., Lugar-Hammer M., Gandjbakhche A., Knutson J.R., Balaban R.S. (2011). Optimizing multi-photon fluorescence microscopy light collection from living tissue by non-contact total emission detection (epiTED). J. Microsc..

[bib4] Dumas J.H., Kinisley S.B. (2005). Two-photon excitation of di-4-ANEPPS for optical recording of action potentials in rabbit heart. Ann. Biomed. Eng..

[bib5] Dvornikov A., Gratton E. (2016). Imaging in turbid media: a transmission detector gives 2-3 order of magnitude enhanced sensitivity compared to epi-detection schemes. Biomed. Opt. Express.

[bib6] Ghouri I.A., Kelly A., Burton F.L., Smith G.L., Kemi O.J. (2015). 2-photon excitation fluorescence microscopy enables deeper high-resolution imaging of voltage and Ca^2+^ in intact mice, rat, and rabbit hearts. J. Biophot..

[bib7] Kelly A., Ghouri I.A., Kemi O.J., Bishop M.J., Bernus O., Fenton F.H., Myles R.C., Burton F.L., Smith G.L. (2013). Subepicardial action potential characteristics are a function of depth and activation sequence in isolated rabbit hearts. Circ Arrhythmia Electrophysiol.

[bib8] Kelly A., Salerno S., Connolly A., Bishop M., Charpentier F., Stølen T., Smith G.L. (2017). Normal interventricular differences in tissue architecture underlie right ventricular susceptibility to conduction abnormalities in a mouse model of Brugada syndrome. Cardiovasc. Res..

[bib9] Kemi O.J., Loennechen J.P., Wisløff U., Ellingsen Ø. (2002). Intensity-controlled treadmill running in mice : cardiac and skeletal muscle hypertrophy. J. Appl. Physiol..

[bib10] Knollmann B.C., Katchman A.N., Franz M.R. (2001). Monophasic action potential recordings from intact mouse heart: validation, regional heterogeneity, and relation to refractoriness. J. Cardiovasc. Electrophysiol..

[bib11] Kobat D., Horton N.G., Xu C. (2011). In vivo two-photon microscopy to 1.6-mm depth in mouse cortex. J. Biomed. Opt..

[bib12] Kulkarni R.U., Kramer D.J., Pourmandi N., Karbasi K., Bateup H.S., Miller E.W. (2017). Voltage-sensitive rhodol with enhanced two-photon brightness. Proc. Natl. Acad. Sci..

[bib13] Larsen A.P., Sciuto K.J., Moreno A.P. (2012). The voltage-sensitive dye di-4-ANEPPS slows conduction velocity in isolated Guinea pig hearts. Heart Rhythm.

[bib14] Leray A., Lillis K., Mertz J. (2008). Enhanced background rejection in thick tissue with differential-aberration two-photon microscopy. Biophys. J..

[bib15] Litovsky S.H., Antzelevitch C. (1988). Transient outward current prominent in canine ventricular epicardium but not endocardium. Circ. Res..

[bib16] Loew L.M. (1996). Potentiometric dyes: imaging electrical activity of cell membranes. Pure Appl. Chem..

[bib17] McMullen J.D., Zipfel W.R. (2010). A multiphoton objective design with incorporated beam splitter for enhanced fluorescence collection. Opt. Express.

[bib18] McPheeters M.T., Wang Y.T., Werdich A.A., Jenkins M.W., Laurita K.R. (2017). An infrared optical pacing system for screening cardiac electrophysiology in human cardiomyocytes. PLoS One.

[bib19] Miller E.W., Lin J.Y., Frady E.P., Steinbach P.A., Kristan W.B., Tsien R.Y. (2012). Optically monitoring voltage in neurons by photo-induced electron transfer through molecular wires. Proc. Natl. Acad. Sci..

[bib20] Myles R.C., Bernus O., Burton F.L., Cobbe S.M., Smith G.L., Rc M., Bernus O., Fl B., Sm C., Effect S.G.L. (2010). Effect of activation sequence on transmural patterns of repolarization and action potential duration in rabbit ventricular myocardium. Am. J. Physiol. Heart Circ. Physiol..

[bib21] Nabauer M., Beuckelmann D.J., Uberfuhr P., Steinbeck G. (1996). Regional differences in current density and rate-dependent properties of the transient outward current in subepicardial and subendocardial myocytes of human left ventricle. Circulation.

[bib22] Nygren A., Kondo C., Clark R.B., Giles W.R. (2003). Voltage-sensitive dye mapping in Langendorff-perfused rat hearts. Am. J. Physiol. Heart Circ. Physiol..

[bib23] Pakhomov A.G., Semenov I., Casciola M., Xiao S. (2017). Neuronal excitation and permeabilization by 200-ns pulsed electric fi eld : an optical membrane potential study with FluoVolt dye. BBA - Biomembr..

[bib24] Paredes R.M., Etzler J.C., Watts L.T., Lechleiter J.D. (2008). Chemical calcium indicators. Methods.

[bib25] Pawlicki M., Collins H.A., Denning R.G., Anderson H.L. (2009). Two-photon absorption and the design of two-photon dyes. Angew. Chem. Int. Ed..

[bib26] Rubart M., Wang E., Dunn K.W., Field L.J. (2003). Two-photon molecular excitation imaging of Ca^2+^ transients in Langendorff-perfused mouse hearts. Am. J. Physiol. Heart Circ. Physiol..

[bib27] Sampson K.J., Henriquez C.S. (2005). Electrotonic influences on action potential duration dispersion in small hearts : a simulation study. Am. J. Physiol. Heart Circ. Physiol..

[bib28] Schaffer P., Ahammer H., Muller W., Koidl B., Windisch H. (1994). Di-4-ANEPPS causes photodynamic damage to isolated cardiomyocytes. Pflugers Arch Eur J Physiol.

[bib29] Singh A., McMullen J.D., Doris E.A., Zipfel W.R. (2015). Comparison of objective lenses for multiphoton microscopy in turbid samples. Biomed. Opt. Express.

[bib30] Smith G., Reynolds M., Burton F., Kemi O.J. (2010). Confocal and multiphoton imaging of intracellular Ca^2+^. Methods in Cell Biology.

[bib31] Stobrawa G., Berchner-Pfannschmidt U., Porwol T., Spiess E., Bestvater F., Acker H., Feurer T., Wotzlaw C., Hacker M. (2002). Two-photon fluorescence absorption and emission spectra of dyes relevant for cell imaging. J. Microsc..

[bib32] Theer P., Denk W. (2006). On the fundamental imaging-depth limit in two-photon microscopy. J. Opt. Soc. Am. A.

[bib33] Thireau J., Zhang B.L., Poisson D., Babuty D. (2008). Heart rate variability in mice: a theoretical and practical guide. Exp. Physiol..

[bib34] Tung C.K., Sun Y., Lo W., Lin S.J., Jee S.H., Dong C.Y. (2004). Effects of objective numerical apertures on achievable imaging depths in multiphoton microscopy. Microsc. Res. Tech..

[bib35] Wen Q., Gandhi K., Capel R.A., Hao G., Shea C.O., Neagu G., Pearcey S., Pavlovic D., Terrar D.A., Wu J., Faggian G., Camelliti P., Lei M., Bers D., Vandenberg J. (2018). Transverse cardiac slicing and optical imaging for analysis of transmural gradients in membrane potential and Ca2+ transients in murine heart. J. Physiol..

[bib36] Woodford C.R., Frady E.P., Smith R.S., Morey B., Canzi G., Palida S.F., Araneda R.C., Kristan W.B., Kubiak C.P., Miller E.W., Tsien R.Y. (2015). Improved PeT molecules for optically sensing voltage in neurons. J. Am. Chem. Soc..

[bib37] Ying J., Liu F., Alfano R.R. (1999). Spatial distribution of two-photon-excited fluorescence in scattering media. Appl. Opt..

[bib38] Young P.A., Clendenon S.G., Byars J.M., Decca R.S., Dunn K.W. (2011). The effects of spherical aberration on multiphoton fluorescence excitation microscopy. J. Microsc..

[bib39] Zhang Y., Kong L., Xie H., Han X., Dai Q. (2018). Enhancing axial resolution and background rejection in line-scanning temporal focusing microscopy by focal modulation. Adv Photonics.

