# Supplementary material for: Two-photon excitation of FluoVolt allows improved interrogation of transmural electrophysiological function in the intact mouse heart
Source: Prog Biophys Mol Biol. 2020 Aug;154:11–20. doi: 10.1016/j.pbiomolbio.2019.08.007 (PMC7322535; doi:10.1016/j.pbiomolbio.2019.08.007)
Supplement: Multimedia component 1 [file mmc1.docx]

**Supplementary materials and methods**

## Axial beam profile imaging

Imaging of the two photon excitation (TPE) profile of a scattering fluorescent sample was performed similar to the method described by Ying et al., (1999).^1^ An ultrafast tunable Ti:Sapphire laser source (Chameleon Ultra, tunable Ti:Sapphire laser; Coherent, Santa Clara CA, US) was used to create a focused excitation spot inside a liquid fluorescent sample of dilute voltage-sensitive fluorophore. The beam was passed through the scan head of an LSM510 microscope (Zeiss; Oberkochen, Germany) with the scanning mirrors parked so the beam entered the centre of the objective lens (10x 0.3NA water dipping, 3.5mm working distance) back aperture. A customised sample chamber was designed using Autodesk Fusion360 (Mill Valley CA, US) and printed in black PLA using a Prusa I3 MK2/S 3D printer (Prusa Research, Czech Republic). The sample chamber was cube-shaped and designed to accommodate size 0 22mm^2^ glass coverslips on two imaging faces. A second objective lens (0.3NA, 10X air, 10 mm working distance) was positioned at right angles to the TPE objective, at one of the two imaging faces. The objective was attached to a 50mm tube lens housing an f25 planoconvex lens and a 650nm shortpass filter to eliminate any stray two-photon excitation light. The 50mm tube was then mounted to a CCD camera (Dalsa CA-0256W, 256^2^ pixel imaging sensor). The lens setup allowed an ~2.5mm^2^ image onto the CCD sensor. An objective lens collar was attached to the top of the chamber, allowing it to be mounted to the TPE objective lens. To view the entire working distance of the TPE objective, a set of images were acquired incorporating the TPE objective focal spot. The microscope z-stage was then moved 1.5mm down and a second set of images was acquired which incorporated the TPE objective lens front aperture. As the sample chamber was mounted to the objective lens, the axial profile remained unchanged and could therefore be reconstructed in full using multiple images. A total of 5 images at each position were captured by the CCD camera at a rate of 500Hz to allow image integration in the case of low signal amplitude. Images were then stitched together using an ImageJ plugin post-experiment. To minimize the contribution of out-of-plane scattered fluorescence on the axial profile images, the beam was focused as close to the coverslip as possible.

The measured two-photon excitation profiles of FluoVolt and di-4-ANEPPS are quite distinct (see Figure 1C of main manuscript) and so the axial profiling experiments were repeated using two different wavelengths; 840 nm, where both spectra overlap; and 1000nm, where FluoVolt excitation is minimal. This reduced the possibility of an excitation wavelength bias.

## Imaging limitations

TPE microscopy normally employs high (>0.8) NA objective lenses to maximise spatial confinement of excitation pulse energy. To resolve the same spot size in the axial imaging profile then requires an objective lens with matched numerical aperture. In practice this is not possible due to physical constraints imposed by (i) the relatively short working distance of the TPE lens (usually only a few mm) and (ii) the physical size of the objective lens barrel, which can extend 3-5 times beyond the distance of the objective lens front aperture. In the end it was found that the smallest TPE objective lens barrel available required the working distance of the axial imaging objective to be at least 9mm to ensure adequate separation between the two objectives. Commercially available objective lenses matching this working distance requirement have NAs of 0.5 at best. Therefore, matching the NA of the axial imaging objective to that of the TPE lens would have required an expensive custom designed lens, without yielding much more significant information from the sample. The original paper by Ying et al (1999) utilized two objective lenses with NA of 0.25. This study was able to adequately predict the major phenomenological features of out-of-focus TPE fluorescence despite the low NA. In practice, using lower than normal NA objective lenses for this study would be predicted simply to overestimate the degree of out-of-focus fluorescence for a given laser power than might be seen using high NA objectives with shorter working distances. No qualitative change in the results would be expected.

## References

## 1 Ying J, Liu F, Alfano RR. Spatial distribution of two-photon-excited fluorescence in scattering media. Appl Opt. 1999;38:224–9

**Supplementary Figures**

**
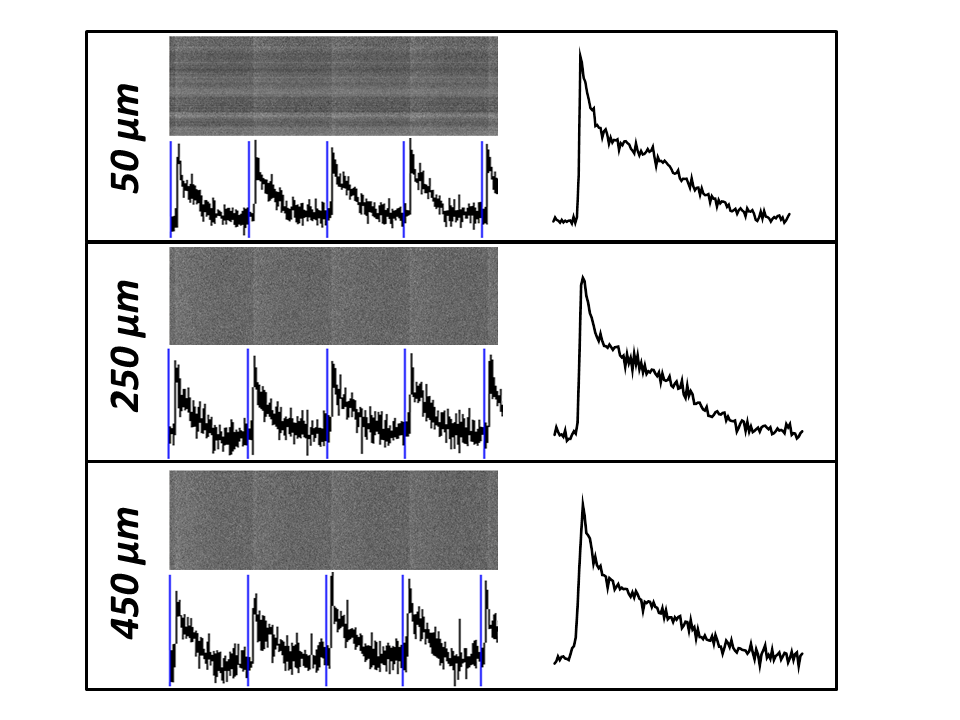
**

**Supplementary Figure 1 – Di-4-ANEPPS line scan images and derived APs with increasing depth**. 5000-6000 lines (rows in the greyscale images – 250-400 pixels per line) were averaged pixel-wise to obtain a train of APs, which was subsequently averaged by pacing cycle length (blue vertical lines) to obtain a single AP (right) with high signal-to-noise for each transmural layer. Horizontal stripes in the 50µm line scan represent surface features (blood vessels) resolved in the uppermost cell layers, which are removed by the averaging process.


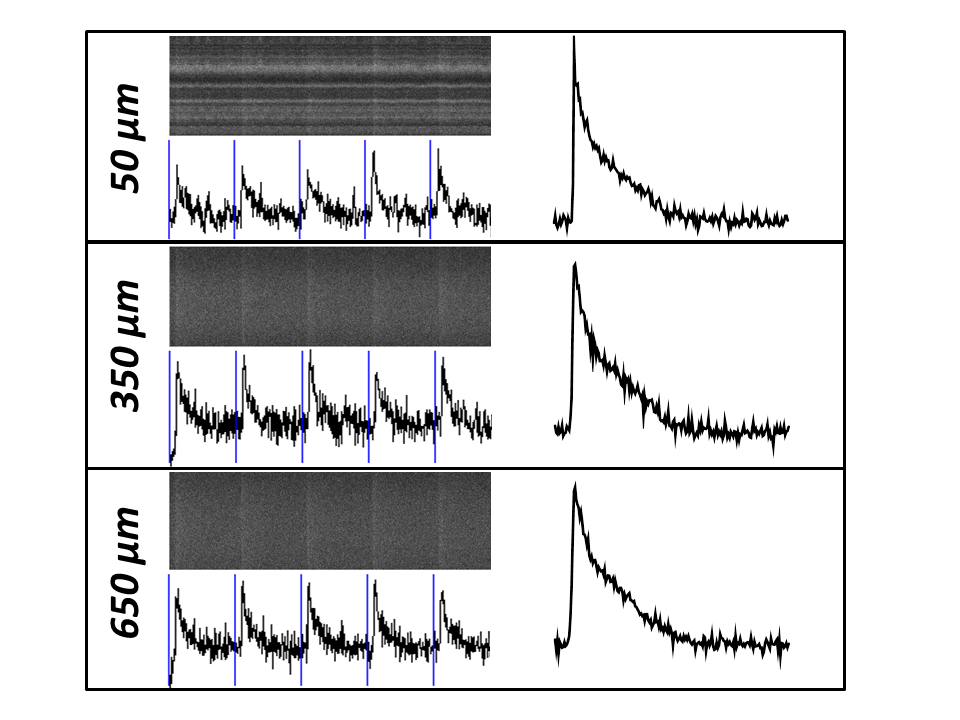


**Supplementary Figure 2 – FluoVolt line scan images and derived APs with increasing depth**. 5000-6000 lines (rows in the images) were averaged pixel-wise to obtain a train of APs, which was subsequently averaged by pacing cycle length (blue vertical lines) to obtain a single AP (right) for each transmural layer. Horizontal stripes in the 50µm line scan derive from blood vessels resolved in the uppermost cell layers.


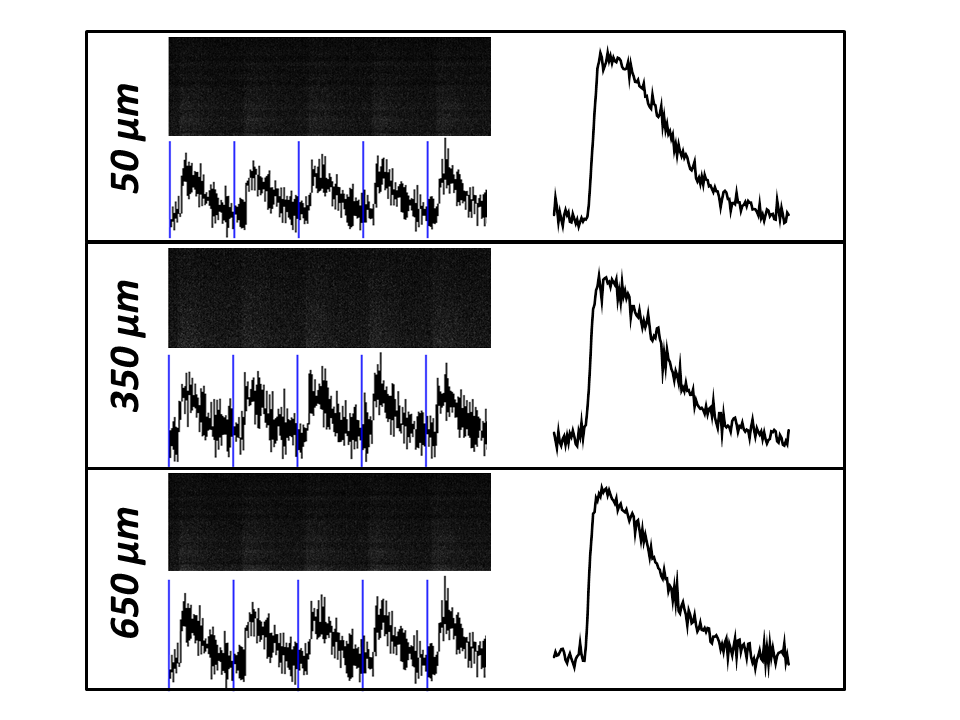


**Supplementary Figure 3 – Rhod2-AM line scan images and derived calcium transients with increasing depth**. 5000-6000 lines (rows in the images) were averaged pixel-wise to obtain a train of calcium transients, which was subsequently averaged by pacing cycle length (blue vertical lines) to obtain a single calcium transient (right) for each transmural layer.

**
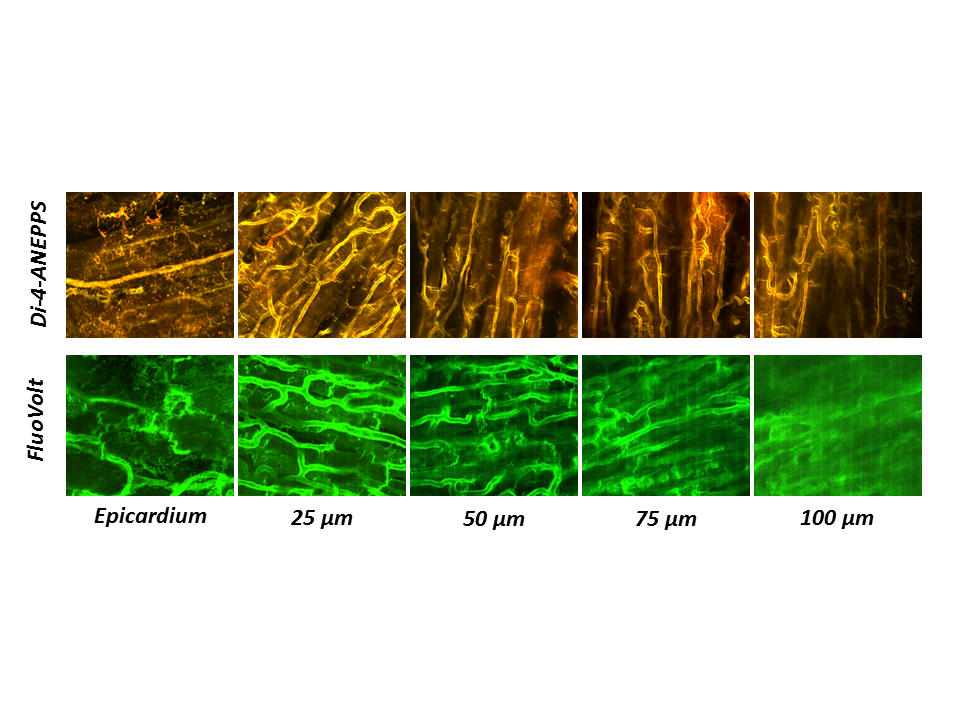
**

**Supplementary Figure 4 – 2D two photon excitation images of Di-4-ANEPPS and FluoVolt loaded myocardium with increasing depth**. Images are contrast enhanced for visual clarity.


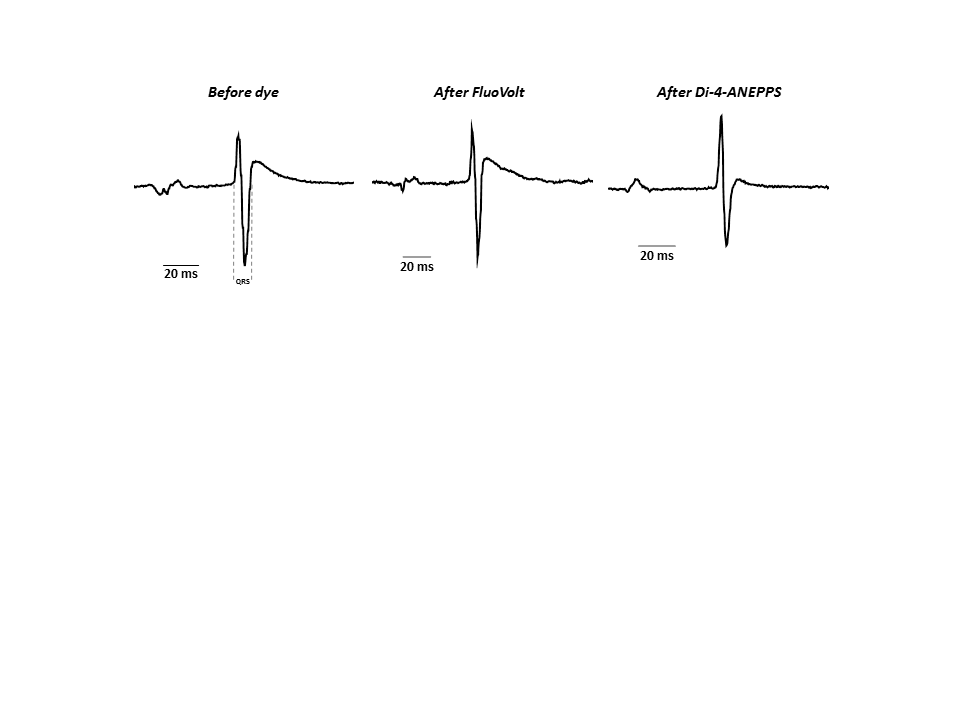


**Supplementary Figure 5 – Representative ECG cycles before and after loading of FluoVolt and di-4-ANEPPS.**


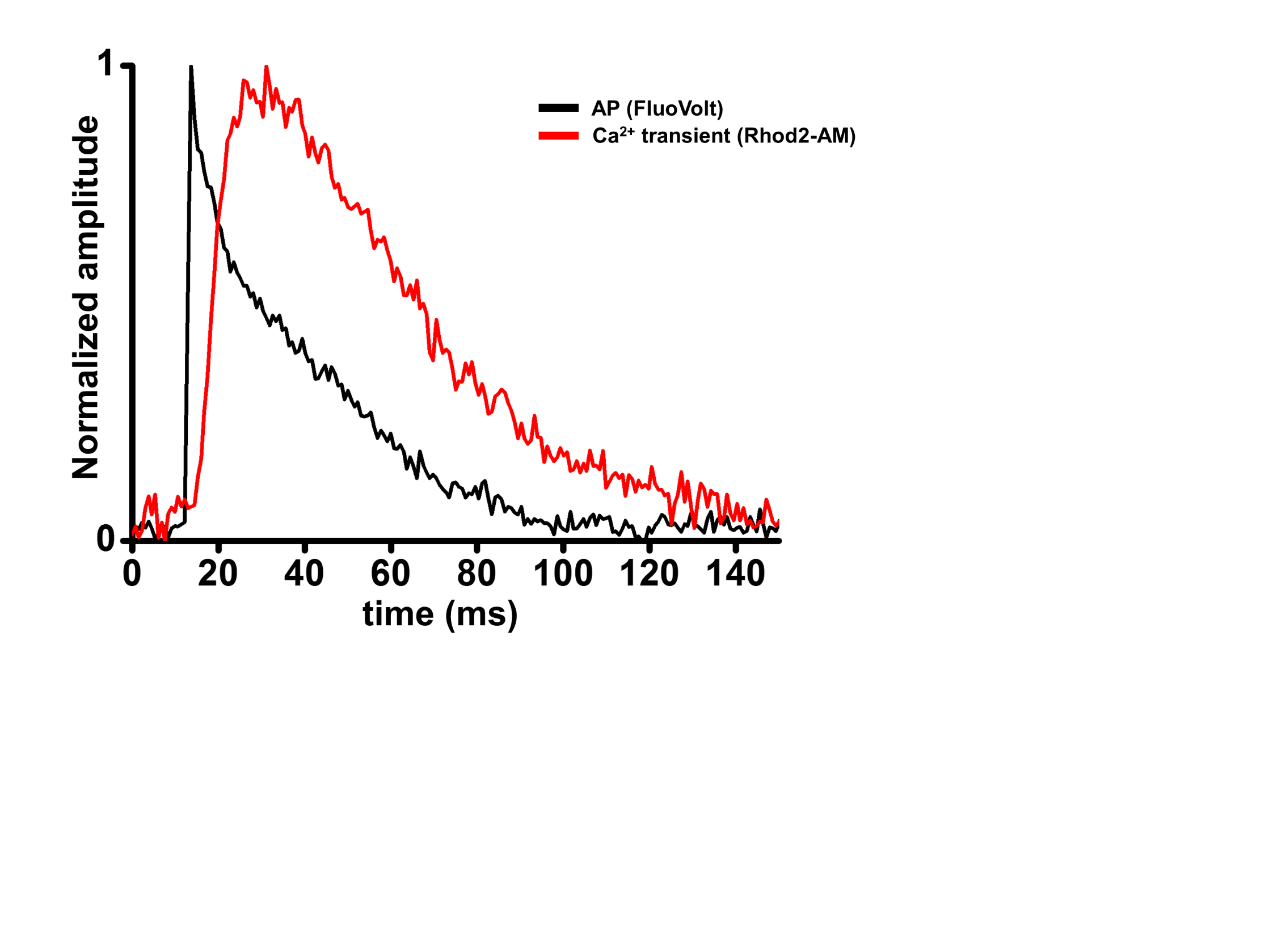


**Supplementary Figure 6 – Simultaneous AP and Ca^2+^ transient acquisition with two-photon excitation**. Typical action potential and intracellular Ca^2+^ transient traces from whole hearts dual-loaded with FluoVolt and Rhod2-AM (excitation 840nm, 50μm below epicardial surface). Simple dual-channel PMT detector setup allows both signals to be recorded with no noticeable cross-talk between emission.


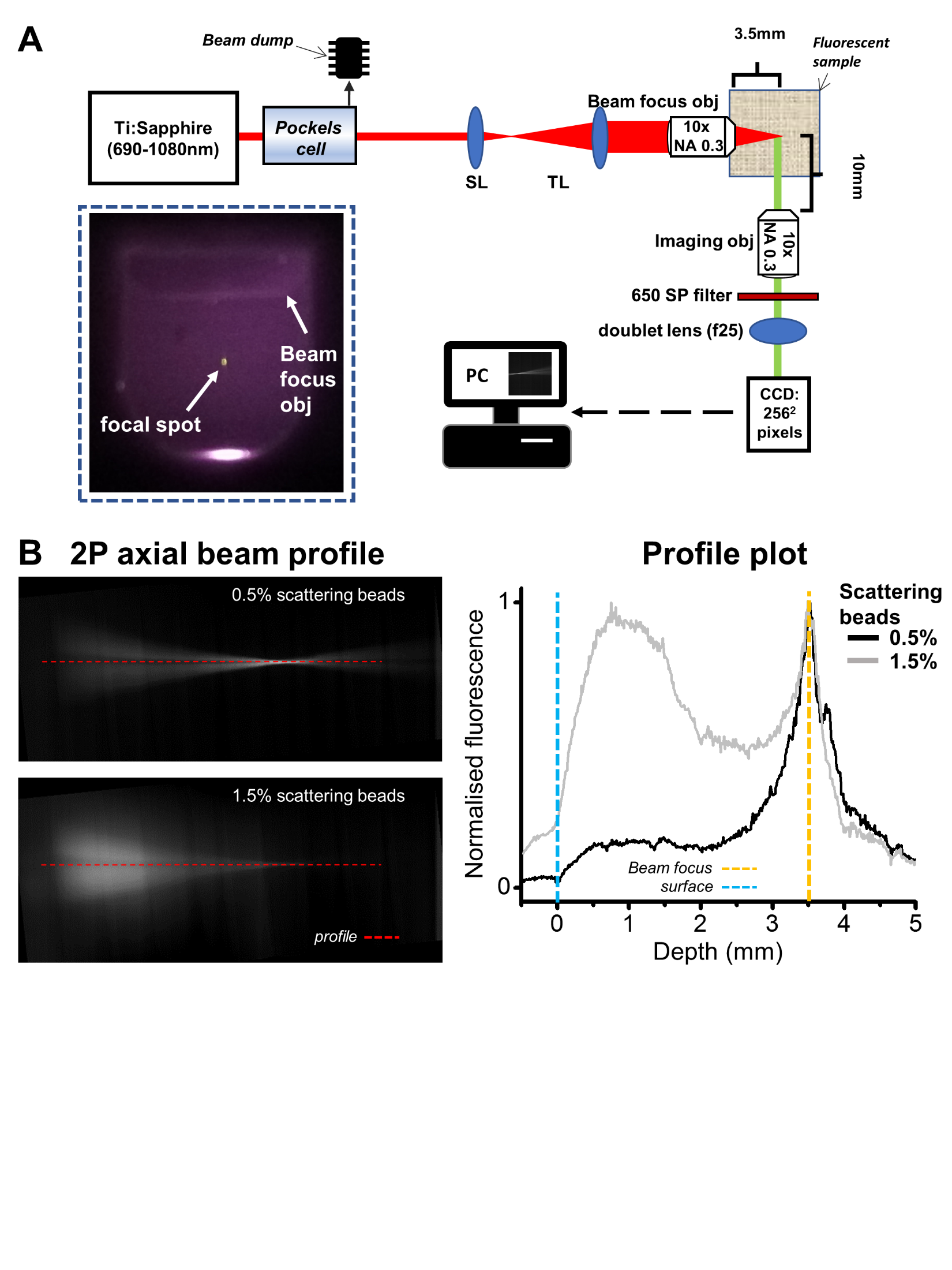


**Supplementary Figure 7 – Assessment of two photon excitation-generated out-of-focus fluorescence.** **(A)** Schematic of imaging setup to measure z-axis two-photon excitation profile in controlled fluorescent samples. Setup based on Ying *et al*., Applied Optics 1999. 38(1):224-229. SL = scan lens; TL = tube lens. Inset – Camera phone image of fluorescent sample in aqueous solution, showing focal spot generated by 0.3NA 10x water-dipping objective. **(B)** ***Left panels -*** Axial beam profile images composed of 3 stitched images (laser beam propagates from left to right). Sample contains indicated concentration of scattering beads to generate out-of-focus fluorescence. Red dash line indicates profile taken through centre of the beam focus. ***Right panel* –** Data from profile indicated in images. Dashed lines indicate objective lens front aperture (OFA, blue) and beam focus (yellow).
